# Phytochemical Analysis and Trypanocidal Activity of *Marrubium incanum* Desr.

**DOI:** 10.3390/molecules25143140

**Published:** 2020-07-09

**Authors:** Claudio Frezza, Alessandro Venditti, Armandodoriano Bianco, Mauro Serafini, Massimo Pitorri, Fabio Sciubba, Maria Enrica Di Cocco, Eleonora Spinozzi, Loredana Cappellacci, Anders Hofer, Filippo Maggi, Riccardo Petrelli

**Affiliations:** 1 Department of Environmental Biology, University of Rome “La Sapienza”, Piazzale Aldo Moro 5, 00185 Rome, Italy; claudio.frezza@uniroma1.it (C.F.); mauro.serafini@uniroma1.it (M.S.); 2 Department of Chemistry, University of Rome “La Sapienza”, Piazzale Aldo Moro 5, 00185 Rome, Italy; alessandro.venditti@gmail.com (A.V.); armandodoriano.bianco@uniroma1.it (A.B.); pitorrimassimo@hotmail.it (M.P.); fabio.sciubba@uniroma1.it (F.S.); mariaenrica.dicocco@uniroma1.it (M.E.D.C.); 3 School of Pharmacy, University of Camerino, via Sant’Agostino 1, 62032 Camerino, Italy; eleonora.spinozzi@unicam.it (E.S.); loredana.cappellacci@unicam.it (L.C.); riccardo.petrelli@unicam.it (R.P.); 4 Department of Medical Biochemistry and Biophysics, Umeå University, 90736 Umeå, Sweden; anders.hofer@umu.se

**Keywords:** *Marrubium incanum* Desr., secondary metabolites, salvigenin, antiprotozoal, Human African Trypanosomiasis (HAT)

## Abstract

The rationale inspiring the discovery of lead compounds for the treatment of human parasitic protozoan diseases from natural sources is the well-established use of medicinal plants in various systems of traditional medicine. On this basis, we decided to select an overlooked medicinal plant growing in central Italy, *Marrubium incanum* Desr. (Lamiaceae), which has been used as a traditional remedy against protozoan diseases, and to investigate its potential against Human African trypanosomiasis (HAT). For this purpose, we assayed three extracts of different polarities obtained from the aerial parts of *M. incanum*—namely, water (MarrInc-H_2_O), ethanol (MarrInc-EtOH) and dichloromethane (MarrInc-CH_2_Cl_2_)—against *Trypanosoma brucei* (TC221), with the aim to discover lead compounds for the development of antitrypanosomal drugs. Their selectivity index (SI) was determined on mammalian cells (BALB/3T3 mouse fibroblasts) as a counter-screen for toxicity. The preliminary screening selected the MarrInc-CH_2_Cl_2_ extract as the most promising candidate against HAT, showing an IC_50_ value of 28 μg/mL. On this basis, column chromatography coupled with the NMR spectroscopy of a MarrInc-CH_2_Cl_2_ extract led to the isolation and identification of five compounds i.e. 1-α-linolenoyl-2-palmitoyl-3-stearoyl-*sn*- glycerol (**1**), 1-linoleoyl-2-palmitoyl-3-stearoyl-*sn*-glycerol (**2**), stigmasterol (**3**), palmitic acid (**4**), and salvigenin (**5**). Notably, compounds **3** and **5** were tested on *T. brucei*, with the latter being five-fold more active than the MarrInc-CH_2_Cl_2_ extract (IC_50_ = 5.41 ± 0.85 and 28 ± 1.4 μg/mL, respectively). Furthermore, the SI for salvigenin was >18.5, showing a preferential effect on target cells compared with the dichloromethane extract (>3.6). Conversely, stigmasterol was found to be inactive. To complete the work, also the more polar MarrInc-EtOH extract was analyzed, giving evidence for the presence of 2″-*O*-allopyranosyl-cosmosiin (**6**), verbascoside (**7**), and samioside (**8**). Our findings shed light on the phytochemistry of this overlooked species and its antiprotozoal potential, providing evidence for the promising role of flavonoids such as salvigenin for the treatment of protozoal diseases.

## 1. Introduction

Human African trypanosomiasis (HAT), also known as sleeping sickness, is triggered by a protozoan parasite named *Trypanosoma brucei* (*T. brucei*), and is one of the 20 neglected tropical diseases (NTDs) that affect low economic status regions in tropical areas. HAT is caused by two parasites from the genus *Trypanosoma* and the species *T. brucei*—namely, *T. brucei gambiense* (*T. b. gambiense*) and *T. brucei rhodesiense* (*T. b. rhodesiense*). *T. b. gambiense* is responsible for about 98% of the cases in humans [1], whereas *T. b. rhodesiense* is accountable for an acute form of trypanosomiasis, characterized by fast disease development and a high mortality rate [2].

The vector fly (tsetse flies of the *Glossina* genus) that transmits HAT to humans is spread in a vast territory of 36 African countries, with the disease prevalent in 19 of them (an area which is also known as the “flies belt”). Within these countries, HAT affects areas from small villages to entire regions. Rural populations who rely on fishing, hunting, agriculture, and animal husbandry are the most exposed. HAT occurs in two stages: the hemolymphatic stage (early-stage infection) and the meningoencephalitic stage (late-stage infection). In the earlystage, the parasite enters the lymphatic system and then passes into the bloodstream (BS) and circulates through the body, causing mild, flu-like symptoms [2]. When the trypanosomes cross the blood-brain barrier (BBB), late-stage infection develops, in which severe neurological complications become apparent. One of them is when the sleep/wake cycle becomes erratic, giving this infectious disease its name.

To date, while we wait for a much-hoped-for vaccine, there are five drugs in our arsenal for HAT treatment: suramin, pentamidine, melarsoprol, eflornithine and nifurtimox [3]. A sixth drug, fexinidazole, that had been in preclinical development as a broad-spectrum anti-infective agent by the Sanofi-Aventis group, recently ended up clinical trials successfully and has now become the first all-oral treatment for HAT [4].

Even though all of these drugs are reasonably efficacious, they suffer from major drawbacks, ranging from the necessity for intravenous administration, which poses a severe problem in rural areas, to stage-specific efficacy as well as relevant toxicity. Even though fexinizadole represents a step forward in terms of administration, safety and efficacy, it necessitates a high pill burden, displays cross-resistance with nifurtimox, and resistance develops quickly *in vitro*, requiring additional research efforts for the discovery of new drug candidates [5,6].

Nowadays, as a result of an intensive campaign of prevention over the past 20 years, the number of reported cases has fallen (<1000 in 2019), deluding people and health services into relaxing their vigilance. However, as long as a vaccine is not available, the drugs are not sufficiently efficient, and people continue to carry the parasite, the tse-tse fly will continue to spread the disease. 

In this challenging scenario, naturally occurring compounds represent a valid and inspiring alternative for developing future trypanocidal drugs [7,8,9,10,11,12]. For difficult-to-reach communities that are extremely exposed to HAT, the use of scientifically validated herbal preparations in the management of the disease ensures the effective and safe use of those remedies and helps to strengthen the healthcare system. The rationale inspiring the discovery of lead compounds for the treatment of human parasitic protozoan diseases from natural sources is the well-established use of medicinal plants in the various systems of traditional medicine. This principle has already led to the discovery of the antimalarial drugs quinine and artemisinin [13,14]. On this basis, we decided to select an overlooked medicinal plant growing in central Italy that has been used as a traditional remedy against protozoan diseases and investigate its potential against HAT.

**Figure 1:**
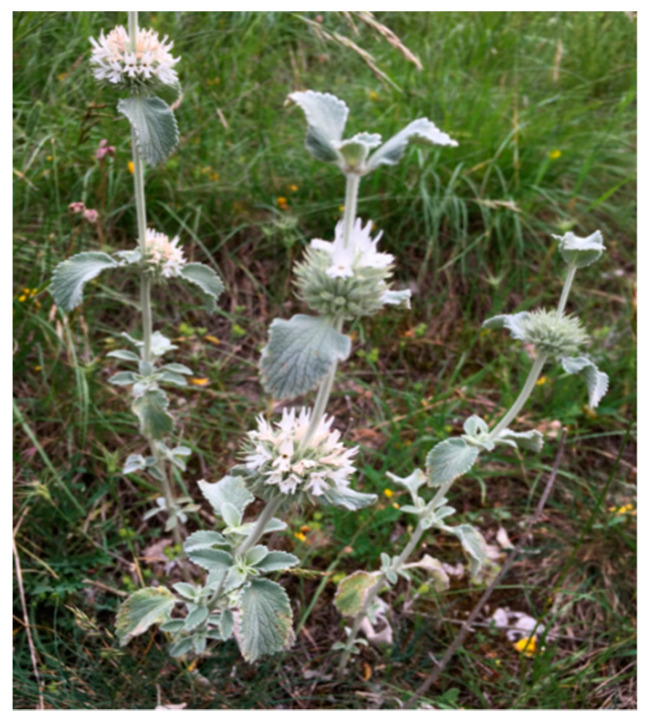
Marrubium incanum Desr.

Belonging to the Lamiaceae family and better known by the common name of white horehound, *Marrubium incanum* Desr. (Figure 1) is a small perennial herbaceous plant native to the Eastern Mediterranean Basin, especially Italy and the Balkan area. The name of the species refers to the presence of thick white hairiness covering the stems and leaves. The plant usually grows on arid and rocky lands up to the altitude of 1200 m a.s.l. [15]. From a botanical point of view, this species is characterized by buds at the ground level and by one stretched floral axis, generally leafless. The plant is endowed with rhizomes, while the erected stem presents a quadrangular section; the leaves are typically opposite and petiolate, and the inflorescences develop as multiple thyrsoid verticillasters intertwined along the stem, with hermaphrodite flowers and a fused corolla; the fruit is a very small schizocarp composed of four nutlets [16].

*M. incanum* is a well-known medicinal plant, especially in the Mediterranean and neighboring regions, showing several therapeutic applications (Table 1). In particular, the population of southern Italy and the native Albanian regions consider *M. incanum* as medicinally equivalent to *M. vulgare* L., due to the same curative uses [17]. The leaf powder is topically effective in treating wounds [18], while the aerial parts are used in decoctions, especially as an appetizer, diuretic and digestive, but also as an antimalarial agent [19] and to treat menstrual pain [16]. The plant is also used for veterinary purposes, to treat foot-and-mouth disease as well as skin disorders through the application of washes [20]. Besides its use in treating stomach diseases, it is also effective for the treatment of heart diseases, such as arrhythmia [18,21]. Finally, its essential oil showed antimicrobial activities [22].

Inspired by the potential of *M. incanum* secondary metabolites to interact with protozoan parasites [17], in the present work we have investigated the phytochemical composition and the effect of *M. incanum* extracts against *T. brucei* spp. To our knowledge, nothing is known about either the antitrypanosomal activity of *M. incanum* extracts or the components responsible for its biological activity. This paper also aims to fill a gap in the literature regarding the complete phytochemical characterization of *M. incanum* extracts.

## 2. Results and Discussion

### 2.1. Phytochemical Analysis

The aerial parts of *M. incanum* collected during the flowering stage in central Italy were dried, pulverized and then extracted by maceration in dichloromethane (MarrInc-CH_2_Cl_2_), ethanol (MarrInc-EtOH) and water (MarrInc-H_2_O).

**Figure 2:**
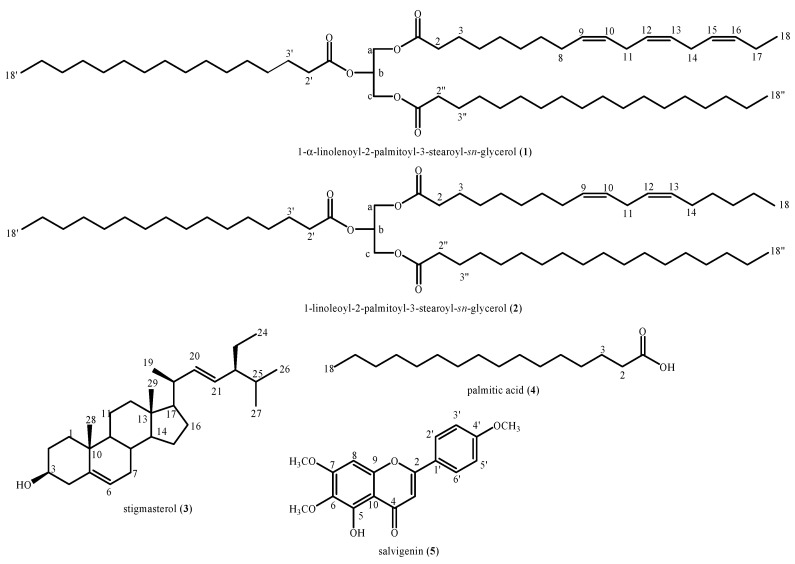
Structures of the compounds identified in the MarrInc-CH_2_Cl_2_ extract.

The phytochemical analysis of the MarrInc-CH_2_Cl_2_ extract led to the isolation and identification of five compounds—i.e., 1-α-linolenoyl-2-palmitoyl-3-stearoyl-*sn*-glycerol (**1**), 1-linoleoyl -2-palmitoyl-3-stearoyl-*sn*-glycerol (**2**), stigmasterol (**3**), palmitic acid (**4**), and salvigenin (**5**) (Figure 2).

**Figure 3:**
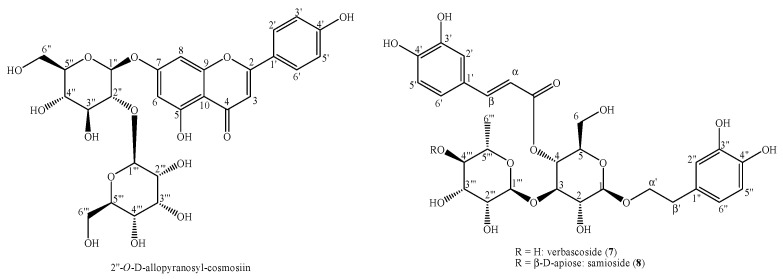
Structures of the compounds identified in the MarrInc-EtOH extract.

In contrast, the phytochemical analysis of the MarrInc-EtOH extract led to the isolation and identification of three compounds—i.e., 2″-*O*-allopyranosyl-cosmosiin (**6**), verbascoside (**7**), and samioside (**8**) (Figure 3).

To the best of our knowledge, stigmasterol (**3**), salvigenin (**5**) and verbascoside (**7**) represent newly isolated compounds from this species, whereas 2″-*O*-allopyranosyl-cosmosiin (**6**) and samioside (**8**) are newly isolated compounds from the genus. Indeed, stigmasterol (**3**) has already been evidenced in the *Marrubium* genus, notably in the species *M. vulgare* [28] and *M. deserti* (Noë) Coss. (now a synonym of *Ballota deserti* (Noë) Jury, Rejdali, and A.J.K. Griffiths) [29]. Salvigenin (**5**) has been already reported in the species *M. thessalum* Boiss. and Heldr. [30], *M. peregrinum* L. [31], *M. cylleneum* Boiss. and Heldr. [32] and *M. velutinum* Sm. [33]. Lastly, verbascoside has been reported in some taxa, such as *M. globosum* subsp. *libanoticum* (Boiss.) P.H.Davis [34], *M. alysson* L. [35], and *M. vulgare* L. [36].The occurrence of these compounds is also well known within the family Lamiaceae [37] and within other families such as Orobanchaceae [38] and Scrophulariaceae [39]. For this reason, these compounds cannot be considered as chemotaxonomic markers at any level due to their wide presence in the plant kingdom.

As far as other secondary metabolites are concerned, 2″-*O*-allopyranosyl-cosmosiin (**6**) and samioside (**8**) have been previously isolated from other species. In particular, compound (**6**), which is a derivative of apigenin with two saccharide residues, has been detected in the Lamiaceae family only in the genus *Sideritis* L. [40,41] and in other families such as Taxaceae [42], Caprifoliaceae [43], and Leguminosae [44]. Compound (**8**), which is a derivative of verbascoside with a further saccharidic residue (β-D-apiose), has been evidenced in the Lamiaceae family in the genus *Phlomis* L. [45] and in *Teucrium chamaedrys* L. [46], as well as in other families such as Orobanchaceae [38] and Verbenaceae [47]. On the above, also these compounds cannot be used as chemotaxonomic markers of *M. incanum*.

Lastly, 1-α-linolenoyl-2-palmitoyl-3-stearoyl-*sn*-glycerol (**1**), 1-linoleoyl-2-palmitoyl-3-stearoyl- *sn*-glycerol (**2**) and palmitic acid (**4**) are primary metabolites constituting the cell wall of all the vegetal organisms and have no chemotaxonomic importance. 

### 2.2. In Vitro Evaluation of the Antiprotozoal Activity of Extracts and Isolated Compounds

Given the traditional use of the plant in Italy as a treatment against protozoal diseases [19], in the present study we assayed three extracts of different polarities obtained from the aerial parts of *M. incanum*—namely, water (MarrInc-H_2_O), ethanol (MarrInc-EtOH) and dichloromethane (MarrInc-CH_2_Cl_2_)—against *T. brucei* (TC221), with the aim to discover lead compounds for the development of antitrypanosomal drugs.

**Figure 4:**
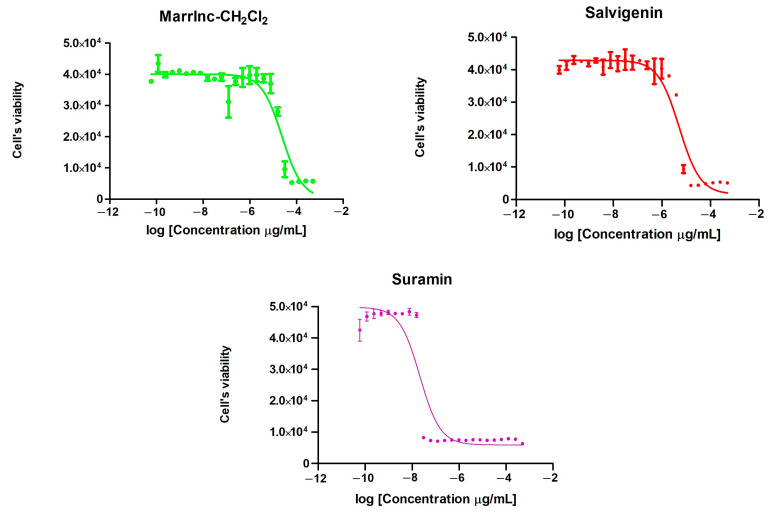
Growth inhibition of *T. b*. bruceiTC221 cells induced by Marrinc-CH_2_Cl_2_ (green), salvigenin (red), and suramin (purple). The graphs show the average results from eightindependent experiments with standard errors.

The three extracts were tested against bloodstream forms of *T. brucei* (TC221) to assess their *in vitro* antitrypanosomal activity, as well as against mammalian cells (BALB/3T3 mouse fibroblasts) as a counter-screen for toxicity (Table 2). This preliminary screening selected the MarrInc-CH_2_Cl_2_ extract as the most promising candidate, which was then subjected to repeated chromatographic separations to yield stigmasterol (**3**) and salvigenin (**5**). Isolated stigmasterol (**3**) and salvigenin (**5**) were also tested against TC221 and BALB/3T3 cells. As reported in Table 2 and Figure 4, salvigenin (**5**) was by far the most active compound, with a five-fold higher activity than the dichloromethane extract but was less active than the reference compound suramin. Furthermore, the selective index (SI) for salvigenin (**5**) was >18.5, showing a preferential effect on the target cells compared with the dichloromethane extract (>3.6). Conversely, stigmasterol (**3**) was found to be inactive. 

Our results clearly showed that the bioactivity of the dichloromethane extract could be, in part, ascribed to the flavonoid salvigenin. It is well known that polyphenols exhibit a wide range of pharmacological activities, ranging from anti-inflammatory [48], antioxidant [49], and anti-proliferative [50] to antiprotozoal effects [51]. 

The different pharmacological activities observed with flavonoids are due to the number and specific position of the phenolic hydroxyl groups, as well as the nature of substituents around the 15-carbon skeleton. The phenolic hydroxyl group at C-5 of salvigenin can easily dissociate to form the negatively charged phenolate ion under physiological conditions, which quickly interacts with several proteins (e.g., enzymes, transcription factors and carriers) by binding them via ionic bonding or hydrogen bonds. This phenomenon leads to a conformational change in the target proteins, with a consequent alteration of their biological activities. Another unique feature of flavonoids, and in this instance of salvigenin, is their ability to switch off several crucial genes in order to prevent viral and parasitic infections [50,52].

Therefore, these hypothesized mechanisms of actions may be behind the good antitrypanosomal activity observed with salvigenin. However, further investigations are sought in order to clarify the mechanism of action of salvigenin and to evaluate its therapeutic value for the treatment of trypanosomiasis infections.

## 3. Materials and Methods 

### 3.1. Chemical and Reagents

The following reagents and solvents were used: dichloromethane, 96% ethanol and distilled water for the extraction procedure of the plant material; *n*-butanol, distilled water, *n*-hexane, ethyl acetate, dichloromethane, and methanol as pure solvents or in mixtures among them at different concentration ratios as eluting systems for the separation procedure by column chromatography; silica gel 60 (70−230 mesh Merck and 200−400 mesh, Merck, Darmstadt, Germany) as a stationary phase for the separation procedure by column chromatography. Thin-layer chromatography (TLC) was carried out on silica gel 60 F254 plates and was employed in order to monitor the column chromatography on-going and assemble fractions having similar retention factors (Rf).Sulfuric acid (2N) was employed to develop all the TLCs: CDCl_3_, CD_3_OD, and D_2_O were used as solvents for the identification of the metabolites by NMR spectroscopy; HPLC methanol was used as a solvent for the identification of the metabolites by MS. All the reagents and solvents were purchased from Sigma-Aldrich Chemical Co (St. Louis, MO, USA), were of analytical grade, if not differently specified, and wereused as received. Silica gel was purchased from Fluka Analytical (Buchs, Switzerland) and TLCs from Merck (Darmstadt, Germany).

### 3.2. Instruments 

The NMR spectra were recorded on a Bruker Avance III 400 MHz instrument (Brucker, Billerica, MA, USA) operating at 9.4 T and 298 K, with the chemical shifts expressed in ppm. The TMS (tetramethylsilane) signal (s, 0 ppm) was used as an internal reference standard for the spectra in CDCl_3_, whereas the internal solvent signal of CD_2_HOD (m5, 3.31 ppm) was the reference for the spectra in CD_3_OD. The chemical shift values are expressed in δ values (ppm), and the coupling constants (*J*) are in hertz. The proton chemical data are reported as follows: chemical shift, multiplicity (s = singlet, d = doublet, dd = doublet of doublets, pd = pseudo doublet, t = triplet, dt = doublet of triplets, pt = pseudo triplet, q = quartet, dq = doublet of quartets, pq = pseudo quartet, m = multiplet, br s = broad singlet), coupling constant(s), integration. The presence of all the exchangeable protons was confirmed by the addition of D_2_O. The MS spectra were instead performed on a Q-TOF MICRO spectrometer (Micromass, now Waters, Manchester, UK) equipped with an ESI source operating in the negative and/or positive ion mode. The flow rate of the sample infusion was 20 μL/min, and 50 acquisitions were made for each analysis. The data were analyzed using the MassLynx software (version 4.1) developed by Waters. The water (H_2_O) was purified by a Milli-Q system (Millipore, Billerica, MA, USA) in our laboratory.

### 3.3. Plant Material

The flowering aerial parts of *M. incanum* (750 g) were collected in Capolapiaggia, Camerino, central Italy (N 43°08′54.21″, E 13°06′38.63″, 650 m a.s.l.) in June 2016. The botanical identification was performed by Domenico Lucarini of the School of Bioscience and Veterinary Medicine, University of Camerino, after the comparison of the morphological data with those reported in the literature [16]. A voucher specimen of the plant is archived in the Herbarium of the School of Biosciences and Veterinary Medicine, University of Camerino, Italy, under the code CAME#27770 and published in the anArchive system for botanical data (an archive system, http://www.anarchive.it).

### 3.4. Preparation of Plant Extracts

The *M. incanum* aerial parts have been air-dried in the shade at room temperature (≈25 °C) for four days and conserved in wrapping papers before extraction. An amount of 350 g of dry aerial parts were reduced into powder using a blender MFC DCFH 48 IKA-WERK (Staufen, Germany) equipped with sieves of 2-mm size in diameter. The powder was subsequently macerated in 5.0 L of dichloromethane (CH_2_Cl_2_) for 48 h in order to allow the extraction of all the present metabolites and filtered. The filtrate was concentrated under a reduced pressure at 30 °C with a rotary evaporator (Buchi, Cornaredo, Italy) and freeze-dried to obtain a crude CH_2_Cl_2_ extract (17.6 g, 4.9% yield, MarrInc-CH_2_Cl_2_). Subsequently, the same amount of powdered plant material (350 gr) was extracted with 5.0 L of ethanol (EtOH) for 48 h in order to allow the extraction of all the present metabolites, yielding 28.8 gr of crude extract (8.2% yield, MarrInc-EtOH). Using the same protocol, an aqueous extract was obtained by the maceration of 10 g of dry material in 100 mL of deionized water to get 0.85 g (8.5% yield, MarrInc-H_2_O) of extract. All the extracts were stored in glass vials protected from light at –20 °C before phytochemical analysis and antitrypanosomal experiments.

### 3.5. Isolation and Identification of Phytochemicals

An aliquot of the dichloromethane extract (MarrInc-CH_2_Cl_2_, 3.0 g) was subjected to a first column chromatography separation step using 90.0 g of silica gel as the stationary phase (ratio 1:30 w/w) and eluting with a solvent gradient of increasing polarity from CH_2_Cl_2_-MeOH 98:2 to 60:40. Since from the first purification, it was not possible to identify any compound, a second chromatographic purification was performed. The combined fractions (1.6 gr) were further subjected to C-18 silica gel column chromatography (ratio 1:35 w/w), eluting with a solvent gradient of increasing polarity from *n*-hexane-EtOAc 9:1 to 2:8. Five compounds were identified in this step, by comparison of their ^1^H NMR spectra (available as Supplementary Materials) with the ^1^H NMR data already reported in literature: 1-α-linolenoyl-2-palmitoyl-3-stearoyl-*sn*-glycerol (**1**, 3.1 mg) as an almost pure compound from the assembly of fractions 1–8 [53,54,55]; 1-linoleoyl-2-palmitoyl-3-stearoyl-*sn*-glycerol (**2**) [53,54,55] and stigmasterol (**3**) [56] in a mixture in the concentration ratio of 1:1 (w/w) from the assembly of fractions 9–15 for the total weight of 4.8 mg; palmitic acid as a pure compound (**4**, 18.6 mg) from the assembly of fractions 46–68 [53,54,55]; salvigenin (**5**, 21.6 mg) as an almost pure compound from the assembly of fractions 95–120 [57].

The ethanol extract (3.0 g) was subjected to a first column chromatography separation step using 100.0 g of silica gel as the stationary phase (ratio 1:33 w/w) and a mixture of *n*-butanol and distilled water (82:18, v/v) as the eluting system. During the chromatographic run, the polarity of the eluting system was raised in order to allow the elution of the most polar constituents passing to a solution composed of *n*-butanol, methanol, and distilled water (70:10:30, v/v/v). From this procedure, three further compounds were identified, by comparison of their ^1^H NMR spectra (available as Supplementary Materials) with the ^1^H NMR data already reported in literature: 2″*O*-allopyranosyl-cosmosiin (**6**) [58] in a mixture with other compounds (ratio not detectable) from the assembly of fractions 5–10 for the total weight of 81.1 mg; verbascoside (**7**) [59] and samioside (**8**) [45] in a mixture in the concentration ratio of 1:10 (w/w) from the assembly of fractions 35–43 (32.2 mg).

*1-α-Linolenoyl-2-palmitoyl-3-stearoyl-sn-glycerol (**1**)*: ^1^H NMR (CDCl_3_, 400 MHz) δ: 5.40–5.33 (6H, m, H-9, H-10, H-12, H-13, H-15, H-16), 5.15–5.12 (1H, m, H-b), 4.29 (2H, dd *J* = 4.3, 12.5 Hz, H-a1, H-c1), 4.14 (2H, dd *J* = 4.3, 12.5 Hz, H-a2, H-c2), 2.81 (4H, t, *J* = 6.4 Hz, H-11, H-14), 2.42–2.27 (6H, m, H-2, H-2′, H-2″), 2.10–2.02 (4H, m, overlapped signals, H-8, H-17), 1.67–1.51 (6H, m, overlapped signals, H-3, H-3′, H-3″), 1.28–1.22 (60H, m, (CH_2_)_n_), 0.93–0.83 (9H, m, overlapped signals, H-18, H-18′, H-18″). ESI-MS: *m*/*z* 879.63 [M + Na]^+^.

*1-Linoleoyl-2-palmitoyl-3-stearoyl-sn-glycerolrol (**2**)*: ^1^H NMR (CDCl_3_, 400 MHz) δ: 5.37–5.33 (4H, m, H-9, H-10, H-12, H-13), 5.14–5.12 (1H, m, H-b), 4.25–4.21 (2H, m, H-a1, H-c1), 4.09–4.04 (2H, m, H-a2, H-c2), 2.81 (2H t, *J* = 6.0 Hz, H-11), 2.42–2.24 (6H, m, H-2, H-2′, H-2″), 2.13–1.97 (4H, m, overlapped signals, H-8, H-15), 1.70–1.53 (6H, m, overlapped signals, H-3, H-3′, H-3″), 1.28–1.22 (66H, m, (CH_2_)_n_), 1.02–0.83 (9H, m, overlapped signals, H-18, H-18′, H-18″). ESI-MS: *m*/*z* 881.51 [M + Na]^+^.

*Stigmasterol (**3**)*: ^1^H NMR (CDCl_3_, 400 MHz) δ: 5.32 (1H, m, H-6), 5.13 (1H, m, H-21), 4.92 (1H, m, H-20), 3.65 (1H, m, H-3), 1.07 (3H, s, H-29), 0.95 (3H, d, *J* = 6.3 Hz, H-19), 0.89 (3H, d, *J* = 6.3 Hz, H-24), 0.87 (3H, d, *J* = 6.2 Hz, H-26), 0.84 (3H, d, *J* = 6.1 Hz, H-27), 0.79 (1H, s, H-28). ESI-MS: *m*/*z* 435.68 [M + Na]^+^. 

*Palmitic acid (**4**)*: ^1^H NMR (CDCl_3_, 400 MHz) δ: 2.34 (2H, t, *J* = 7.5 Hz, H-2), 1.67–1.59 (2H, m, H-3), 1.35–1.20 (24H, m, (CH_2_)_n_), 0.88 (3H, t, *J* = 6.8 Hz, H-18). ESI-MS: *m*/*z* 255.15 [M − H]^−^. 

*Salvigenin (**5**)*: ^1^H NMR (CDCl_3_, 400 MHz) δ: 7.89 (2H, d, *J* = 8.4 Hz, H-2′, H-6′), 6.88 (2H, d, *J* = 8.4 Hz, H-3′, H-5′), 6.61 (1H, s, H-3), 6.55 (1H, s, H-8), 3.97 (3H, s, 6-OMe), 3.93 (3H, s, 7-OMe), 3.90 (3H, s, 4′-OMe). ESI-MS: *m/z* 329.36 [M+H]^+^; *m/z* 351.36 [M+Na]^+^; *m*/*z* 327.18 [M − H]^−^. 

*2″-O-Allopyranosyl-cosmosiin (**6**)*: ^1^H NMR (CD_3_OD, 400 MHz) δ: 7.98 (2H, d, *J* = 8.9 Hz, H-2′, H 6′), 6.89 (2H, d, *J* = 8.9 Hz, H-3′, H-5′), 6.63 (1H, s, H3), 6,48 (1H, br s, H-6), 6.45 (1H, br s, H-8), 5.08 (1H, d, *J* = 7.3 Hz,H-1″), 4.98–4.81 (1H, overlapped with solvent signal, H-1‴), 3.94–3.35 (overlapped saccharidic signals). ESI-MS: *m*/*z* 616.91 [M + Na]^+^.

*Verbascoside (**7**)*: ^1^H-NMR (CD_3_OD, 400 MHz) δ: 7.66 (1H, d, *J* = 16.2 Hz, H-β), 7.33 (1H, br s, H-2′), 7.14 (1H, br d, *J* = 8.3 Hz, H-6′), 7.00 (1H, d, *J* = 8.3 Hz, H-5′), 6.84 (1H, partially overlapped, H-6″), 6.61 (1H, br s, H-2″), 6.55–6.51 (1H, overlapped signal, H-5″), 6.38 (1H, d, *J* = 16.2 Hz, H-α), 5.31 (1H, d, *J* = 1.8 Hz, H-1‴), 4.28 (1H, d, *J* = 7.8 Hz, H-1), 4.12–3.32 (m, overlapped saccharidic signals), 2.84–2.82 (1H, m, H-β′), 1.03 (3H, d, *J* = 7.1 Hz, H-6‴). ESI-MS: *m*/*z* 646.93 [M + Na]^+^.

*Samioside (**8**)*: ^1^H NMR (CD_3_OD, 400 MHz) δ: 7.60 (1H, d, *J* = 15.9 Hz, H-β), 7.08 (1H, d, *J* = 1.8 Hz H-2′), 6.97 (1H, dd, *J* = 8.3/1.8 Hz, H-6′), 6.80 (1H, d, *J* = 8.3 Hz, H-5′), 6.71 (1H, d *J* = 2.0 Hz, H-2″), 6.70 (1H, d, *J* = 8.1 Hz, H-5″), 6.58 (1H, dd, *J* = 8.1/2.0 Hz, H-6″), 6.29 (1H, d, *J* = 15.9 Hz, H-α), 5.23 (1H, br s, H-1^IV^), 5.17 (1H, d, *J* = 2.2 Hz, H-1‴), 4.46 (1H, d, *J* = 7.8 Hz, H-1), 4.12–3.32 (m, overlapped saccharidic signals), 2.81–2.78 (2H, m, H-β′), 1.11 (3H, d, *J* = 7.1 Hz, H-6‴). ESI-MS: *m*/*z* 778.96 [M + Na]^+^.

### 3.6. Cell Culture

Cell culture conditions for *T. brucei* TC221 blood-stream forms and mammalian Balb/3T3 fibroblasts (ATCC no CCL-163) were as described previously [7]. Briefly, *T. brucei* cells were cultured at 37°C with 5% CO_2_ in HMI-9 medium supplemented with 5% (v/v) heat-inactivated fetal bovine serum (Gibco, Billings, MT, USA) [60]. The mammalian fibroblasts were cultured with the same temperature and CO_2_ conditions as the trypanosomes, but the medium was in this case Dulbecco’s modified Eagle’s medium (Sigma-Aldrich) supplemented with 10% (v/v) heat-inactivated fetal bovine serum and L-glutamine (0.584 g/L). In addition, both media contained 10 mL/L of 100× penicillin-streptomycin (Gibco, Billings, MT, USA).

### 3.7. Growth Inhibition Experiments

The plant extracts and compounds were dissolved in dimethyl sulfoxide (DMSO) and serially diluted in growth medium in 96-well plates and mixed with cells (20000 cells/well) to cover a final concentration range from 10^−5^ to 100 μg/mL cell extract or compound. The total volume was 200 µL. After 48 h of incubation under cell culture conditions, 20 μL of 0.5 mM resazurine (Sigma-Aldrich, St. Louis, MO, USA) was added and the cells were incubated for an additional 24 h. The cell density was quantified by the fluorescence at 590 nm (excitation at 540 nm) using an Infinite M200 microplate reader (Tecan Group, Ltd., Männedorf, Switzerland). The IC_50_ values were calculated by the GraphPad Prism 5.04 software on the data plotted on a log inhibitor vs. response curve (variable slope, four parameters).

## 4. Conclusions

The present work provides new insights into the phytochemical and biological properties of *M. incanum.* In particular, eight compounds were isolated, five from the dichloromethane extract—i.e., 1-α-linolenoyl-2-palmitoyl-3-stearoyl-*sn*-glycerol (**1**), 1-linoleoyl-2-palmitoyl-3-stearoyl-*sn*-glycerol (**2**), stigmasterol (**3**), palmitic acid (**4**), and salvigenin (**5**); and three from the ethanol extract—i.e., 2”-*O*-allopyranosyl-cosmosiin (**6**), verbascoside (**7**),and samioside (**8**). Compounds **3, 5** and **7** had never been previously reported in the species, whereas compounds **6** and **8** had never been previously reported in the genus. Their presence confirms the correct botanical identification of the species as an entity belonging to the *Marrubium* genus and the Lamiaceae family, since they have already been isolated from both taxa. Indeed, the exhibited trypanocidal activity of salvigenin (**5**), on the one hand, confirmed the key role played by flavonoids in the treatment of protozoal diseases, and on the other hand, makes it a potential drug candidate for the treatment of trypanosomiasis in developing countries with poor medical infrastructure and severe economic difficulties. Our findings also showed that the traditional use of the plant decoction against protozoal diseases has no scientific background.

## Figures and Tables

**Table 1:** Traditional medicinal uses of *Marrubium incanum*.

Region	Plant Parts	Formulation	Traditional Uses	References
Marche (Italy)	flowers	tincture or infusion	stimulant, depurant of blood, emmenagogue, for losing weight, effective in liver colic	[23,24,25]
Basilicata (Italy; Albanian and Italian communities)	stem, aerial parts	decoction	panacea, in particular as an appetizer digestive, diuretic, antimalarial, against cysts	[19,26,27]
Southern Bosnia and Herzegovina	aerial parts	decoction	treatment of stomach diseases	[21]

**Table 2:** Antitrypanosomal activity of *Marrubium incanum* (MarrInc) extracts and pure compounds.

	IC_50_		Selectivity Index (SI)
	T. b. brucei (TC221)	Balb3T3	
**Extracts**	**μg/mL(μM)**	**μg/mL(μM)**	
MarrInc-EtOH	>100	>100	
MarrInc-H_2_O	>100	>100	
MarrInc-CH_2_Cl_2_	28 ± 1.4	>100	>3.6
**Pure compounds fromMarrInc-CH_2_Cl_2_**	**μg/mL(μM)**	**μg/mL(μM)**	
Stigmasterol (**3**)	>100	>100	
Salvigenin (**4**)	5.41 ± 0.85 (16.43)	>100	>18.5
**Reference drug**	**μg/mL(μM)**	**μg/mL(μM)**	
Suramin	0.0191 ± 0.002 (0.0147)	n.d. ^a^	

^a^ n.d.: not determined.

## Supplementary Materials

The following are available online. ^1^H NMR spectra of compounds 1–8.
