# Supplementary material for: Phytochemical Analysis and Trypanocidal Activity of Marrubium incanum Desr
Source: Molecules. 2020 Jul 9;25(14):3140. doi: 10.3390/molecules25143140 (PMC7397158; doi:10.3390/molecules25143140)
Supplement: Supplementary file 1 [file molecules-25-03140-s001.pdf]

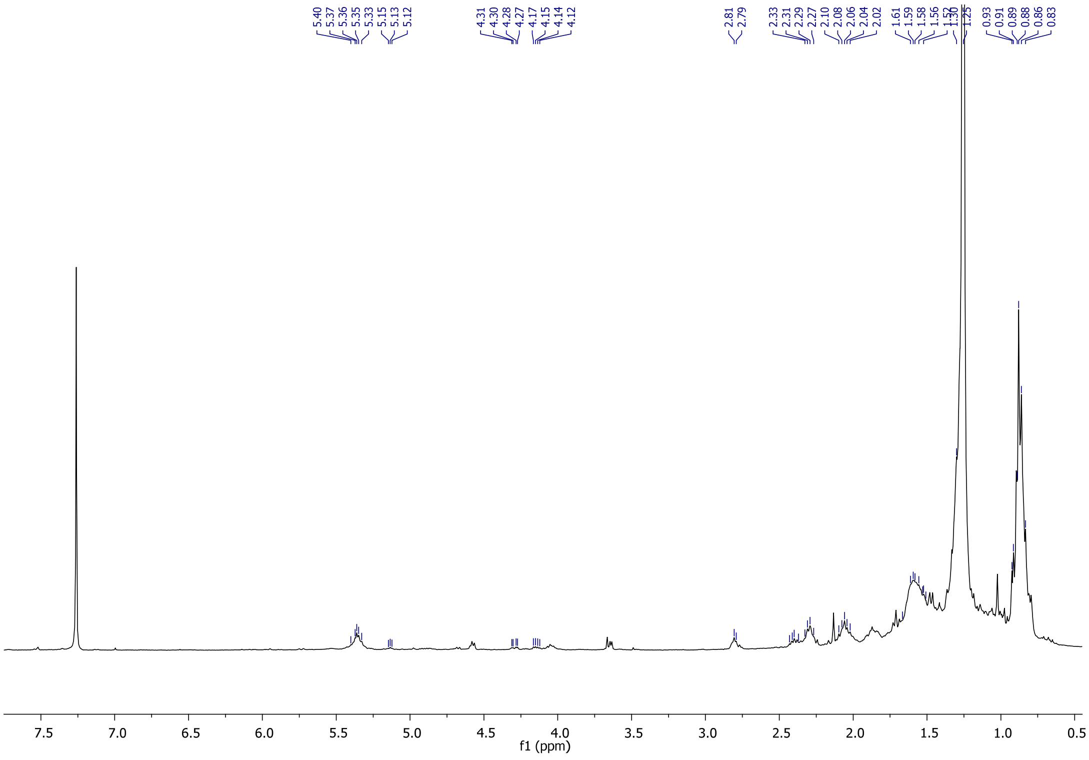

^{1}H NMR spectrum of compound (**1**)

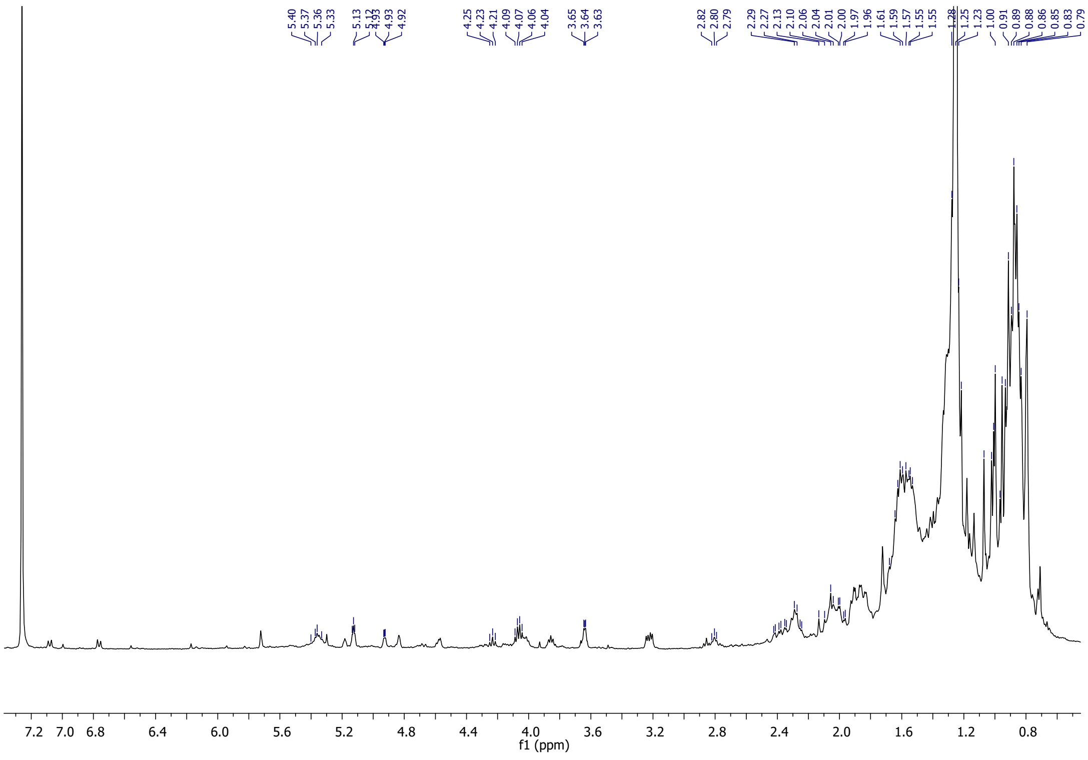

^{1}H NMR spectrum of compounds (**2, 3**)

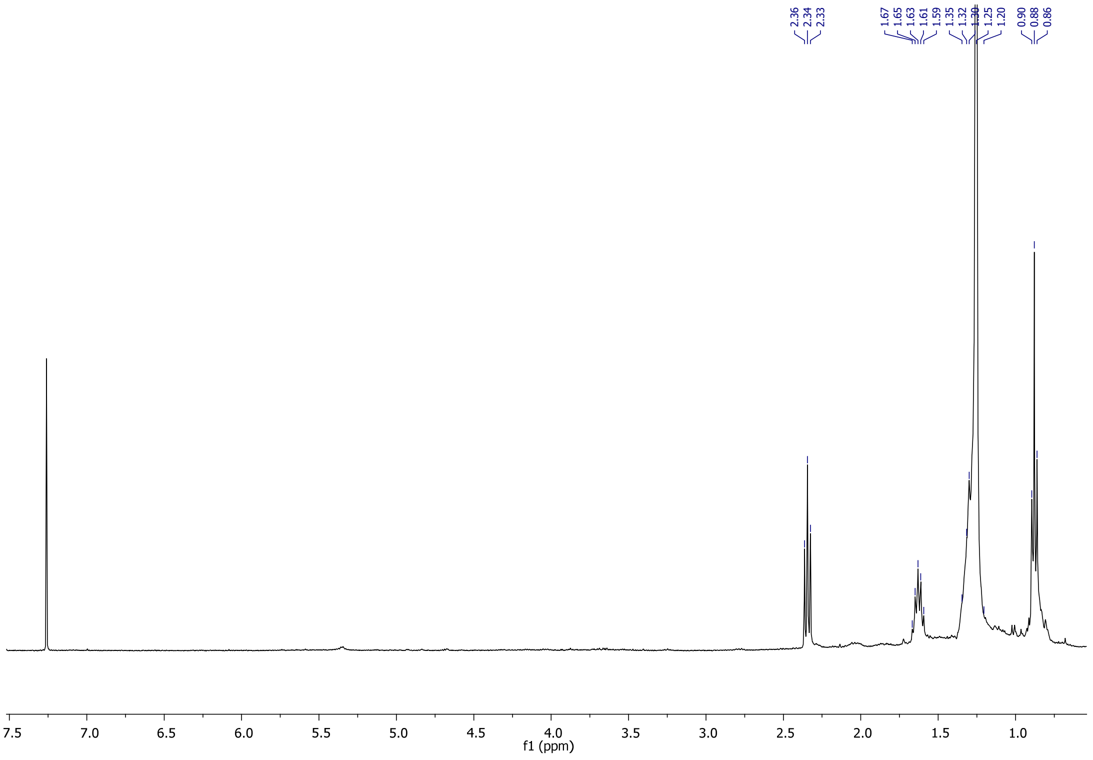

^{1}H NMR spectrum of compound (**4**)

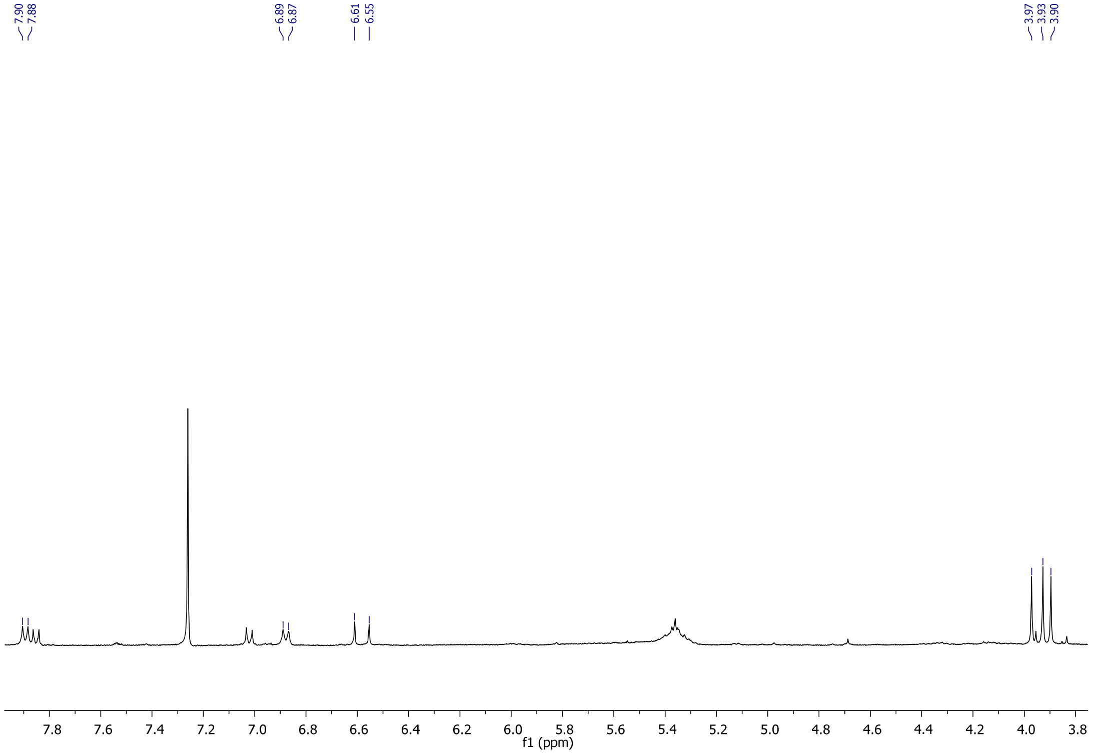

^{1}H NMR spectrum of compound (**5**)

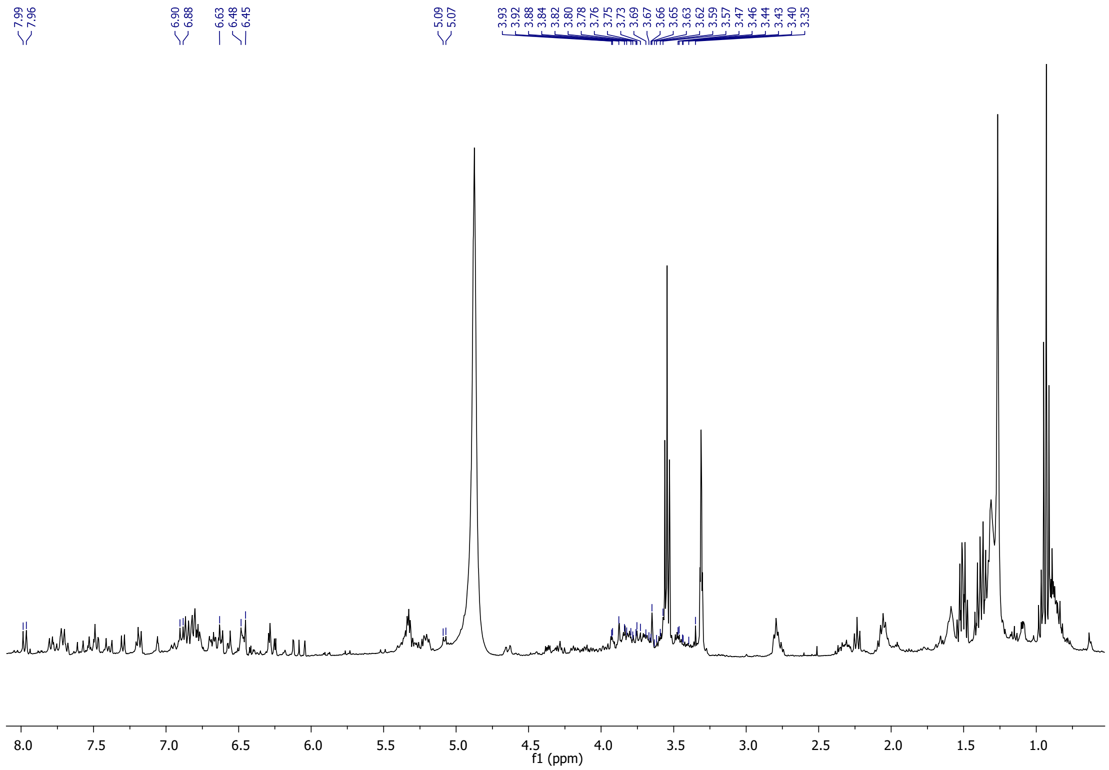

^{1}H NMR spectrum of compound (**6**) and others not identified

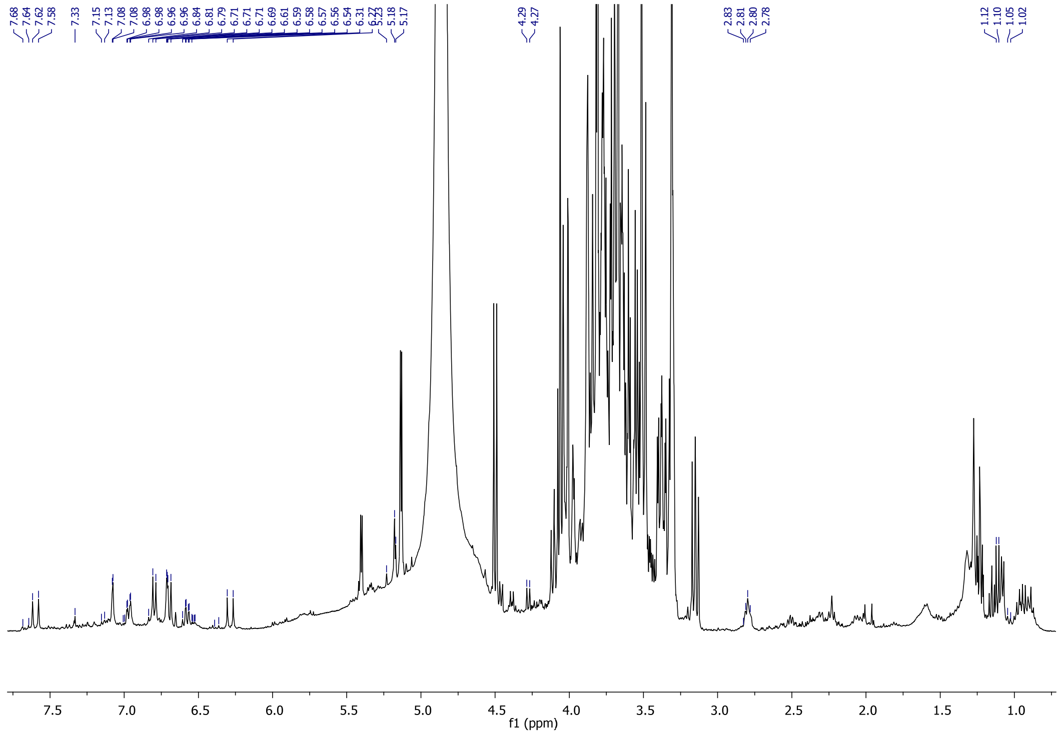

^{1}H NMR spectrum of compounds (**7, 8**)
